# Calcium-Polyphosphate Submicroparticles (CaPP) Improvement Effect of the Experimental Bleaching Gels’ Chemical and Cellular-Viability Properties

**DOI:** 10.3390/gels9010042

**Published:** 2023-01-04

**Authors:** Mariángela Ivette Guanipa Ortiz, Juliana Jarussi dos Santos, Jonny Burga Sánchez, Ubirajara Pereira Rodrigues-Filho, Flávio Henrique Baggio Aguiar, Klaus Rischka, Débora Alves Nunes Leite Lima

**Affiliations:** 1 Department of Restorative Dentistry, Piracicaba Dental School, University of Campinas—UNICAMP, Piracicaba 13414-903, Brazil; 2 Group of Chemistry of Hybrid and Inorganic Materials (GQMATHI), São Carlos Institute of Chemistry, University of São Paulo (USP), São Carlos 13563-120, Brazil; 3 Department of Physiological Science, Piracicaba Dental School, University of Campinas—UNICAMP, Piracicaba 13414-903, Brazil; 4 Fraunhofer Institute for Manufacturing Technology and Advanced Materials IFAM, Wiener Straße 12, 28359 Bremen, Germany

**Keywords:** hydrogen peroxide, bleaching, cell survival, pH, polyphosphates, submicroparticles

## Abstract

The aim of this research was to develop and characterize the chemical and cellular-viability properties of an experimental high-concentration bleaching gel (35 wt%-H_2_O_2_) containing calcium-polyphosphate particles (CaPP) at two concentrations (0.5 wt% and 1.5 wt%). The CaPP submicroparticles were synthesized by coprecipitation, keeping a Ca:P ratio of 2:1. The CaPP morphology, size, and chemical and crystal profiles were characterized through scanning and transmission electron microscopy, energy-dispersive X-ray analysis, and X-ray diffraction, respectively. The assessed bleaching gels were experimental (without CaPP); 0.5% CaPP; 1.5% CaPP; and commercial. The gels’ pH values and H_2_O_2_ concentrations (iodometric titration) were determined. The odontoblast-like cell viability after a gel’s exposure was assessed by the MTT assay. The pH and H_2_O_2_ concentration were compared through a repeated-measures analysis of variance (ANOVA) and a Tukey’s test and the cell viability through a one-way ANOVA and a Tukey’s test using a GraphPad Prism (α < 0.05). The CaPP particles were spherical (with Ca and P, 135.7 ± 80.95 nm size) and amorphous. The H_2_O_2_ concentration decreased in all groups after mixing (*p* < 0.001). The 0.5% CaPP resulted in more-stable pH levels and higher viability levels than the experimental one (*p* < 0.05). The successful incorporation of CaPP had a positive impact on the bleaching gel’s chemical and cellular-viability properties when compared to the experimental gel without these particles.

## 1. Introduction

Given the high levels of dissatisfaction (30%) with teeth darkening, bleaching gels based on hydrogen peroxide have been extensively introduced into the market [1]. Their chief mechanism of action is the oxidation of the organic molecules, which stain teeth, into smaller and lighter compounds [2]. Along with their bleaching effect, both the H_2_O_2_ and its reactive oxygen species (ROS) are responsible for the “nonspecific” oxidative action on the organic and inorganic structural components of enamel [3,4,5,6,7] and dentin [8], generating topographic and mineral changes in these structures, even applying more-neutral or -alkaline gels [9,10,11].

Although enamel is a biologically inert biomaterial, remineralizing components that dissolve through a chemical process can generate local precipitation of biosimilar Ca/PO_4_^3−^ on the surface. This must be considered for the development of dental materials with remineralizing properties, such as bleaching gels, that can achieve an adequate bleaching effect while reducing the intrinsic adverse effects of the therapy [3,12,13,14].

Therefore, the demand for experimental bleaching agents that can ensure maximum benefit with minimum harm has grown in recent years [5,14,15,16,17]. Those that incorporate a source of Ca/PO_4_^3−^ compounds still report changes in the tooth mineral structure [18], and some of the positive evidence could be attributed not only to the compound effect but also to pH changes [14,17] or the decrease in peroxide concentration [1]. For this, it can be inferred that there is still no gold standard Ca/PO_4_^3−^ compound for bleaching-gel preparation.

One promising material is polyphosphate (PolyP), a natural compound of living cells and a source of ortho-phosphate (PO_4_^3−^), which is required for hard tissue formation [19,20]. More recently, the synthesis of calcium-polyphosphate (CaPP) submicroparticles by a defined Ca:P molar ratio of 2:1 has been described [21]. CaPP is an amorphous source of Ca and PO_4_^3−^ that stimulates mineral deposition in both enamel and dentin [19,22]. The interaction of CaPP with these tissues can be partially explained by the formation of Ca^2+^ bonds between the mineral matrix of dental hydroxyapatite and CaPP [19,23].

In view of bleaching treatment–induced mineral alterations and given CaPP’s possible remineralizing effects, the incorporation of CaPP in experimental bleaching gels is an innovative and promising alternative for treatment. Thus, the aim of this research was to develop and characterize the chemical and cellular-viability properties of an experimental high-concentration bleaching gel (H_2_O_2_/HP—35 wt%) containing calcium-polyphosphate (HP-CaPP) particles in two concentrations (0.5 wt% and 1.5 wt%). For this study, the null hypotheses were as follows: (1) the H_2_O_2_ concentration and pH levels of the HP-CaPP bleaching agent will not differ from those of a commercial bleaching agent and an experimental bleaching gel without CaPP, and (2) the cytotoxic potential of the HP-CaPP bleaching agent will not differ from that of a commercial bleaching agent or that of an experimental bleaching gel without CaPP.

## 2. Results

### 2.1. Scanning Electron Microscopy (SEM) and Energy-Dispersive X-ray Analysis (EDX)

**Figure 1:**
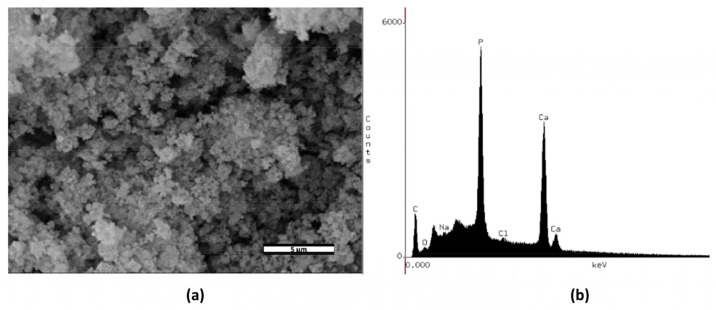
Morphological and chemical characterization of the synthesized calcium-polyphosphate particles: (**a**) scanning electron microscopy (SEM) images (×5000); (**b**) energy-dispersive X-ray analysis (EDX).

SEM analysis showed that the particles had a spherical morphology (Figure 1a). The presence of Ca and P was detected by using the EDX system. Although certain levels of Na and Cl persisted, the amounts were minimal. The particles presented a Ca:P atomic ratio of 1.11, as shown in Figure 1b.

### 2.2. Transmission Electron Microscopy (TEM) and Dynamic Light Scattering (DLS)

**Figure 2:**
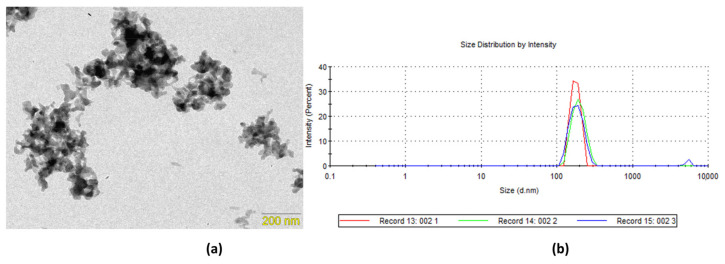
Sized and size distribution of the synthesized calcium-polyphosphate particles: (**a**) transmission electron microscopy (TEM); (**b**) dynamic light scattering (DLS).

According to the TEM images, the CaPP particles showed a submicro particle size (135.7 ± 80.95 nm; Figure 2a). The hydrodynamic radius of the CaPPs showed a distribution of 257.1 nm (±26.52 nm), with a polydispersity index of 0.43 (±0.05) and a correlation of 0.90 (Figure 2b).

### 2.3. X-ray Diffraction (XRD)

**Figure 3:**
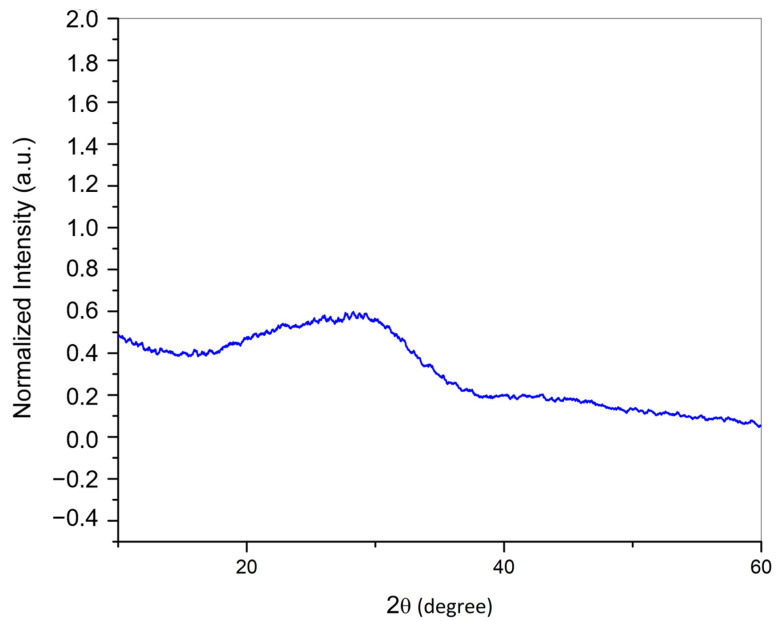
Results of X-ray diffraction analysis (XRD) of the calcium-polyphosphate particles.

XRD analysis demonstrated the amorphous character of the synthesized CaPP particles given that the characteristic peaks of crystalline materials were not observed in the diffractogram (Figure 3).

### 2.4. Visual and pH Evaluation Results

**Figure 4:**
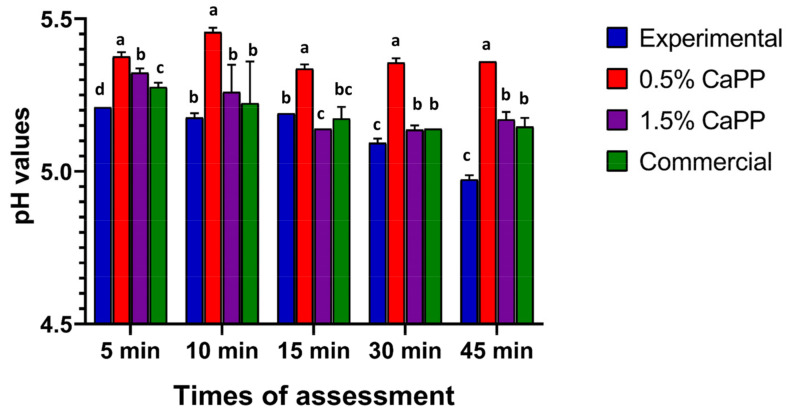
The pH mean values of the used bleaching gels in the different periods of assessment. Different letters in the bars indicate statistically significant differences among the groups (*p* ≤ 0.05). The results were obtained through triplicate analyses.

The visual aspect of the experimental gels (Figure S1) denoted an opaque appearance with increasing CaPP concentrations. The pH values (without dilution) varied among the periods of time (ANOVA; *p* < 0.0001) and among the bleaching gels (ANOVA; *p* < 0.0001). The gel with 0.5% CaPP had the highest pH values when compared to the others. After 15 min of mixing, all gels maintained pH values above 5.1. The 1.5% CaPP and commercial gels (Tukey; *p* < 0.05) had the lowest pH values in this assessment period. After 45 min of mixing, the experimental bleaching gel had the lowest pH value (Tukey; *p* ≤ 0.007) (Figure 4).

### 2.5. Hydrogen Peroxide Concentration of the HP-CaPP—Titrimetric Analysis (Iodometric)

The H_2_O_2_ concentration decreased with time in all groups (ANOVA; *p* < 0.0001). There were no significant differences among the groups for H_2_O_2_ concentration after 15, 30, and 45 min of gel mixing. After 90 min, the experimental bleaching gel presented the lowest H_2_O_2_ concentration (Tukey; *p* < 0.005); however, after 150 min, the 0.5% and 1.5% CaPP gels presented the highest H_2_O_2_ levels (Tukey; *p* < 0.005) (Table 1).

### 2.6. Odontoblasts-like Cells (MDPC-23) Viability after Exposure to the HP-CaPP Bleaching Gel

**Figure 5:**
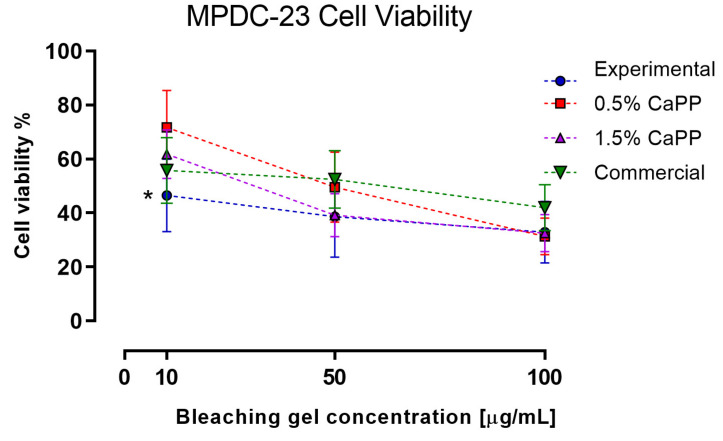
Cell viability (%) of the MDPC-23 cells after exposure to different concentrations of the bleaching gels (10, 50, and 100 µg/mL). * Statistically different from the rest of the groups (*p* < 0.05).

The cell viability results are displayed in Figure 5. For the 10 µg/mL concentration, the experimental gel was significantly different from the other gels (*p* < 0.05; Tukey’s post hoc ANOVA test). The IC_50_ of this experimental gel (9.81) was significantly different from the 0.5% CaPP (35.92), 1.5% CaPP (22.65), and commercial (26) gels. Additionally, the IC_50_ of the 0.5% CaPP was significantly different from the 1.5% CaPP (*p* < 0.05).

## 3. Discussion

An experimental bleaching gel based on H_2_O_2_-35 wt% with calcium-polyphosphate submicroparticles (HP-CaPP) was developed, and its chemical (pH and H_2_O_2_ concentration) and biological (cellular-viability) properties were assessed. According to the obtained results, the first null hypothesis was rejected, as the HP-CaPP bleaching gels (CaPP—0.5 and 1.5 wt%) had higher pH levels compared to the experimental gel without CaPP and similar values to the commercial bleaching gel. The H_2_O_2_ concentration was similar among the bleaching gels. The second hypothesis was also rejected, as the cell viability was higher for the HP-CaPP groups (CaPP—0.5 and 1.5 wt%) than for the experimental gel without CaPP but was similar to the commercial bleaching gel.

Following the coprecipitation method [21], CaPPs were synthetized and characterized for later incorporation into an experimental bleaching gel (HP-CaPP). The SEM images display particles with spherical morphology. Additionally, the EDX spectra display marginal Na levels but significant levels of P and Ca, as previously reported [21,22,24].

Concerning the CaPP size, the TEM analysis indicated submicrometric particles (135.7 ± 80.95 nm). The starting Ca:P molar ratio was 2:1, which allowed the formation of smaller particles [22]. Although the DLS results display a higher hydrodynamic radius (257.1 ± 26.52 nm), this analysis considers the particles’ hydrodynamic diameters (257.1 ± 26.52 nm). Another factor that may influence DLS analysis and contribute to a higher hydrodynamic radius reading is the particle agglomeration that usually occurs throughout the reading [25,26]. As determined by XRD analysis, the CaPPs retained their amorphous character. The amorphous state is relevant for increasing the remineralizing potential of the particles. This is related to the fact that when dental tissues are exposed to highly supersaturated phosphate solutions (e.g., CaPP), an amorphous calcium phosphate precipitate can be formed and later transformed into an organized crystal apatite structure [22,23,27].

The initial pH values of the experimental formulations were intended to remain close to those of the undiluted commercial bleaching gel to determine the effect of the CaPP particles within a similar pH range [28]. The smallest CaPP concentration (0.5 wt%) seemed to have a beneficial effect on pH value stability. Initially, the medium protons, thanks to their high electronegativity, replace the Ca^2+^ ions on the CaPP structure, leading to an increase in pH, as observed after 10 min in the 0.5% CaPP gel. Moreover, phosphoric acid is also produced during the hydrolytic cleavage of the CaPP chain, which decreases the gel’s pH [29]. When we tripled the CaPP concentration (1.5 wt%), this latter effect was predominant, as after 15 min, this gel had a lower pH than the experimental and 0.5% CaPP gels. The commercial gel showed a pH decrease 15 min after mixing (5.17), which followed the trend of previous assessments [15,30].

After the longest assessment time, 45 min after the preparation of the experimental gel without CaPP, the lowest pH values were reached. The lower pH values have been related to higher enamel alterations (e.g., lower microhardness, higher superficial roughness) and to reduced bleaching efficacy [5,7]. Thus, the incorporation of CaPP into an experimental bleaching gel could be regarded as positive for the pH of the bleaching gels and therefore positive for the enamel properties in future in vitro/in vivo applications.

The H_2_O_2_ concentration was lower than 35 wt% 15 min after mixing the gels. However, given the H_2_O_2_ instability, the exact concentration is difficult to determine by titration [31], which can be considered a limitation of the current study. After gel preparation, the H_2_O_2_ present in the bleaching gel starts to decompose into free radicals and H_2_O as time passes [31]. Nevertheless, in the experimental gel containing CaPP, this reduction was not as accentuated as in the experimental gel without CaPP and the commercial gel. This could occur because of the H_2_O_2_ stabilization promoted by the phosphate molecules released from the CaPP chain [31,32,33,34].

Given that CaPP is a biocompatible compound, its presence in bleaching gels, as expected, did not increase their cytotoxic potential [21,35,36]. Interestingly, lower amounts of CaPP (0.5 wt%) were better at sustaining MDPC-23 cells’ viability compared to the experimental gel without CaPP. CaPP chain cleavage not only provides ortho-phosphate units that aid in the remineralization process but also generates “metabolic fuel” by supporting ATP production [21]. This intracellular burst of energy could increase cell proliferation [37,38], which could explain why this group presented the best results for cell viability at a concentration of 10 µg/mL.

However, a higher polyphosphate concentration within the bleaching gel (1.5 wt%) did not result in an increase in cell viability. The hydrolytic cleavage of the CaPP chain led to a more acidic gel, as seen in our pH assessments. Under these conditions, cell growth, proliferation, and differentiation were retarded [39]. It could also be speculated that H_2_O_2_ decomposition into free radicals at this pH level is reduced; therefore, the free remaining H_2_O_2_ cytotoxic potential could have affected cell viability [40,41], surpassing the CaPP protective effect displayed at a lower CaPP concentration and a higher pH level (0.5% CaPP).

As previously described, CaPP, as a long-chain bioinorganic polymer, is a promising bioactive compound in bioremineralization [19,23,42]. This characteristic behavior would be useful to reduce the enamel mineral loss associated with high-concentration bleaching gels, while the presence of the ions would have a potential effect on reducing dental sensitivity, which remains the most common clinical symptom of the bleaching treatment [1,5]. Although the successful incorporation of CaPP did not negatively alter the experimental gels’ chemical and biological properties compared to commercial and experimental bleaching gels based on 35% H_2_O_2_ without these particles, further studies should be conducted to determine the effect of CaPP on the bleaching efficacy and the enamel chemical and mechanical properties of this type of experimental bleaching gel when compared to commercially established treatments.

## 4. Conclusions

In the present study, calcium-polyphosphate fine particles were successfully synthesized through the coprecipitation method [21] and incorporated into bleaching gels based on 35 wt% H_2_O_2_. The incorporation of CaPP, even in smaller concentrations (0.5% w/t), stabilized the pH, reduced the cytotoxic potential, and retained similar hydrogen peroxide concentration values compared to the experimental bleaching gel without CaPP after 45 min of mixture in vitro. Because the CaPP gel’s retained similar chemical and biological properties to the commercial bleaching gel without CaPP, their remineralizing potential turns them into a promising clinical alternative to conventional bleaching gels.

## 5. Materials and Methods

### 5.1. Synthesis of Calcium-Polyphosphate Particles (CaPP) and Characterization

To obtain CaPP particles, a coprecipitation technique was used [21]. Based on our own pilot synthesis and previous works [19,21], a sodium polyphosphate (NaPP), with an average chain length of ~35 phosphate units (Chemische Fabrik; Budenheim, Germany), was used for CaPP synthesis (Table 2).

**Figure 6:**
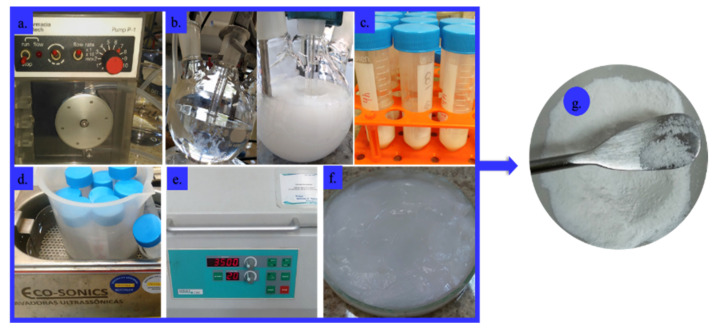
Graphical summary of the synthesis procedure of calcium-polyphosphate (CaPP) particles employing the coprecipitation method: (**a**) flow pump; (**b**) controlled addition of CaCl_2_⋅2H_2_O solution (1 mL/min; pH = 10) into the NaPP solution; (**c**) After 4 h of stirring, distribution of the crude product in falcon tubes; (**d**) washing of CaPP (twice with water and twice with ethanol); (**e**) Centrifugation after every wash; (**f**) CaPP slurry obtained; (**g**) CaPP dried fine powder.

The pH of the NaPP solution (5 g in 250 mL H_2_O) was adjusted to 10 using an aqueous 1 M NaOH solution. Then, the CaCl_2_·2H_2_O (Sigma-Aldrich, Taufkirchen, Germany) solution (14 g in 250 mL H_2_O) was added to the NaPP solution at a controlled rate, 1 mL/min, using a peristaltic pump (P-1; Pharmacia Biotech, Uppsala, Sweden) to obtain a Ca:P ratio of 2:1. During the addition, the pH was maintained (10) at room temperature using 1 M NaOH solution. After 4 h of stirring, the final solution was washed for 10 min and centrifuged (3500 rpm × 20 min) twice with distilled water and at last twice with absolute ethanol. The final slurry was kept in an oven at 60 °C (14 h) [21,22]. The dried material was crushed with a pestle in a mortar to obtain a fine CaPP powder (Figure 6)**.**

#### 5.1.1. Scanning Electron Microscopy (SEM) and Energy-Dispersive X-ray Analysis (EDX)

A thin gold layer was deposited on the CaPP surface (BAL-TEC SCD 050; Capovani Brothers Inc., NY, USA) to assess CaPP morphology. Images at ×5000 magnification were obtained on an SEM (JSM-5600, JEOL; Tokyo, Japan) with a 15 kV accelerating voltage, 13 mm Z, and 15 mm WD.

The powder was coated with carbon by vapor deposition (Delton vacuum Desk II, Moorestown, NJ, USA) to characterize the chemical elements of the CaPP particles. Elemental analysis was performed using an EDX detector (Vantage, Acquisition Engine Company, Tokyo, Japan) connected to an SEM (JSM-5600, JEOL, Tokyo, Japan). The EDX system was operated at 15 kV with a collection time of 100 s, 30° incidence, Z = 20 mm, and WD = 20 mm. Three areas of approximately 10 µm^2^ were evaluated.

#### 5.1.2. Transmission Electron Microscopy (TEM) and Dynamic Light Scattering (DLS)

Morphological and size examinations of CaPP were performed using a transmission electron microscope (Phillips CM 200, Phillips, Amsterdam, the Netherlands) at 200 kV with a LaB_6_ filament. The sample was prepared, and the obtained images were analyzed to estimate the particle size on the basis of an average diameter of 60 particles (ImageJ; public domain image software).

To determine the particle hydrodynamic radius, CaPP was diluted in distilled water (1 mg/mL), stirred for 15 min (50 °C), and centrifuged for 10 min (10,000 rpm). The supernatant was analyzed using a laser diffraction particle analyzer (Zetasizer Nano ZS90; Malvern Instruments Ltd., Worcestershire, UK) with the following parameters: 10 runs of 10 s, at 25 °C, with 60 s of stabilization, and a 90° scattering angle.

#### 5.1.3. X-ray Diffraction (XRD)

The CaPP powder crystallinity pattern was recorded on a diffractometer (D8 Advance; Bruker, MA, USA) with Cu-Kα radiation (40 kV, 30 mA) and an equipment geometry of 2θ. Continuous readings were performed with a step of 0.02° and an accumulation time of 0.3 s (interval of 4°/min).

### 5.2. Experimental Bleaching Gel Containing Calcium-Polyphosphate Particles (HP-CaPP)

Formulations of high-concentration H_2_O_2_-based bleaching gel (H_2_O_2_—35 wt%) with two separate components were proposed: A—H_2_O_2_ (pH ≈ 1.8); B—thickener and calcium-polyphosphate particles (CaPP) (pH ≈ 12) [5]. Three types of experimental bleaching gels were manipulated: without CaPP (experimental) and containing 0.5 and 1.5 wt% CaPP (CaPP 0.5% and CaPP 1.5%). In addition, Whiteness HP Maxx (FGM Dental Products, SC, Brazil) was used as a commercially available control.

The reagents of component A were stirred for 60 min (≈10 °C) in the dark to prevent H_2_O_2_ decomposition [31,43]. For the gels containing CaPP in component B, CaPP was first diluted in distilled water and stirred in a water bath for 15 min (50 °C). After cooling down, the remaining reagents of component B were homogenized (Speed Mixer DAC 150.1; FlackTek, Inc., SC, USA) at 2000–2500 rpm for 10 min (Table 3).

For all gel analyses, experimental components A and B were mixed in a 3:1 weight proportion, while the commercial control bleaching gel was prepared according to the manufacturer’s instructions. All gels were kept under refrigeration (8 ± 1 °C) before the experiment and were brought to room temperature 30 min before mixing.

#### 5.2.1. Evaluation of pH

The pH values of 4 g of each bleaching gel were recorded using a benchtop digital pH meter (mPA210; MS TECNOPON, Piracicaba, SP, Brazil), which was previously calibrated with buffered solutions (pH 4, 7, and 10) at 25 °C. The pH values of the gels were recorded in triplicate 5, 10, 15, 30, and 45 min after the mixture of both components. The mean of the three measurements was considered the final value for each assessment time.

#### 5.2.2. Hydrogen Peroxide Concentration of the HP-CaPP—Titrimetric Analysis (Iodometric)

The gels’ respective H_2_O_2_ concentrations were determined by employing iodometric titration with a hydrogen peroxide test kit (Hanna Instruments; Barueri, SP, Brazil) and a standardized sodium thiosulfate solution (Na_2_S_2_O_3_; 0.1 N). In brief, iodide ions (I^–^) can be oxidized by H_2_O_2_ to iodine (I_2_), which in the presence of starch forms a blue charge-transfer complex by the imprisonment of polyiodide species within the helix structure of amylose. I_2_ can be reduced by titration with a standard Na_2_S_2_O_3_ solution to I^–^, which is colorless [31,44]. The H_2_O_2_ concentration can be calculated by the volume of the standard Na_2_S_2_O_3_ solution required to change the color of the bleaching solution using Equation (1) [45].

(1) $$\;C=\;\frac{\left[\left(0.1M\times V\right)/2\right]\times 34.0147g/mol}{V_{f}}\;\;\;$$

where *C* = final concentration; *V* = volume of the Na_2_S_2_O_3_ (0.1 N) solution used; and *V*_f_ = final volume of the diluted H_2_O_2_ solution used to conduct the titration.

All bleaching gels were diluted in distilled water (1 mg/mL of H_2_O_2_) and remained at room temperature in static and dark conditions. At defined times, samples were collected from the upper area of the samples (15, 30, 45, 90, and 150 min after mixing) and further diluted to obtain 0.2 mg/mL H_2_O_2_ solutions in three 25 mL glass vials [31]. The titration procedure was performed on the three samples, and the mean was considered the final value of each sample/time assessed.

#### 5.2.3. Odontoblasts-like Cells (MDPC-23) Viability in the HP-CaPP Bleaching Gel

The MTT reduction method was performed to determine the viability of MDPC-23 cells after exposure to the bleaching gels. The MDPC-23 cells were cultured in Dulbecco’s modified Eagle’s medium (DMEM, Sigma-Aldrich, St. Louis, MO, USA) supplemented with antibiotics, 100 IU/mL penicillin and 100 μg/mL streptomycin (Vitrocell Embriolife, Campinas, Brazil), 2 mmol/L glutamine, and 10% fetal bovine serum (FBS—GIBCO; Thermo Fisher Scientific, Waltham, MA, USA) at 37 °C in a 5% CO_2_ atmosphere [39,46].

After reaching 80% confluence, the MDPC-23 cells were washed with 0.25% trypsin/EDTA (GIBCO; Thermo Fisher Scientific, Waltham, MA, USA) to separate them from the plate. The separated cells were centrifuged at 3000 rpm for 5 min at 4°C. A Neuberger chamber was used to count the cells. The supernatant was discarded; the cells were passed to a new medium (DMEM), transferred to 96-well cell culture plates (Corning Costar Corp., Cambridge, MA, USA) at a concentration of 5 × 10^4^ cells/mL, and subsequently incubated in a 5% CO_2_ atmosphere at 37 °C for 24 h.

After the incubation period, the cells were exposed to diluted bleaching gels (10, 50, and 100 µg/mL) with DMEM for 45 min. The wells were then washed twice with phosphate buffered saline (PBS—pH = 7.4), and 200 μL of MTT diluted in DMEM medium at 0.3 mg/mL (Invitrogen; Thermo Fisher Scientific, Waltham, MA, USA) was added. After 3 h of incubation in a 5% CO_2_ atmosphere at 37 °C, the wells were washed twice with PBS and filled with 200 μL of ethanol. Finally, the absorbance values were obtained using a microspectrophotometer (ASYS UVM340; Biochrome Ltd., Cambridge, England) at 570 nm [39,47].

### 5.3. Statistical Analyses

Shapiro–Wilk and Levene tests were used to verify the normality and homoscedasticity of variances of the cell viability, pH, and concentration data. The cell-viability data were compared through a one-way analysis of variance (ANOVA) and a post hoc Tukey’s test. The IC_50_ values were determined by logistic regression analysis. The respective pH values and concentrations were assessed through a repeated-measures ANOVA and a post hoc Tukey’s test (significance level 5%) using GraphPad Prism 8.0.2 software for Windows (GraphPad Software, San Diego, CA, USA).

## Figures and Tables

**Table 1:** Hydrogen peroxide concentration of the bleaching gels determined through the iodometric method.

Gel	15 min	30 min	45 min	90 min	150 min
	Hydrogen Peroxide Concentration Mean (wt%) *				
Experimental	26.74 a	25.28 a	21.39 a	17.01 b	14.10 c
0.5% CaPP	25.16 a	24.67 a	23.19 a	22.69 a	23.68 a
1.5% CaPP	24.91 a	24.91 a	23.25 a	23.50 a	21.85 a
Commercial	29.86 a	23.08 a	22.10 a	22.10 a	18.79 b

Different letters in the columns indicate statistically significant differences among the groups (*p* ≤ 0.05). * The results were obtained through triplicate analyses.

**Table 2:** Main reagents used for the synthesis of calcium-polyphosphate (CaPP).

Reagent	Brand	Lot
CaCl_2_⋅2H_2_O (C3306-1000g)	Sigma	#SLBZ8395
Na-PolyP (DP-PCB/O-2018-001)	Chemische Fabrik Budenheim	MV58/371
NaOH (Pearls)	Labsynth	H2000.01.AH

**Table 3:** Composition of the experimental bleaching gels (components A and B).

Component A	Component B
H_2_O_2_ (50% sol)	H_2_O distilled
Carbopol 940	CaPP (0.5 or 1.5 wt%)
Glycerol	Carbopol 940
Propylene glycol	Glycerol
Citric acid	Propylene glycol
-	NaPP
NaOH to adjust the pH ≈ 1.8	NaOH to adjust the pH ≈ 12

## Data Availability

The data that support the findings of this study are available from the corresponding author upon reasonable request.

## Supplementary Materials

The following supporting information can be downloaded at https://www.mdpi.com/article/10.3390/gels9010042/s1, Figure S1: Bleaching gels appearance after manipulation from left to right—experimental; 0.5% CaPP; 1.5% CaPP; commercial.
