# Supplementary material for: Calcium-Polyphosphate Submicroparticles (CaPP) Improvement Effect of the Experimental Bleaching Gels’ Chemical and Cellular-Viability Properties
Source: Gels. 2023 Jan 4;9(1):42. doi: 10.3390/gels9010042 (PMC9857579; doi:10.3390/gels9010042)
Supplement: Supplementary file 1 [file gels-09-00042-s001.zip › gels-2104491-supplementary.pdf]

gels

Article

Calcium-Polyphosphate Submicroparticles (CaPP) Improvement Effect of the Experimental Bleaching Gels' Chemical and Cellular-Viability Properties

Mariángela Ivette Guanipa Ortiz [1], Juliana Jarussi dos Santos [2], Jonny Burga Sánchez [3], Ubirajara Pereira Rodrigues-Filho [2], Flávio Henrique Baggio Aguiar [1], Klaus Rischka [1,4,*], and Débora Alves Nunes Leite Lima [1]

[1] Department of Restorative Dentistry, Piracicaba Dental School, University of Campinas—UNICAMP, Piracicaba 13414-903, SP, Brazil
[2] Group of Chemistry of Hybrid and Inorganic Materials (GQMATHI), São Carlos Institute of Chemistry, University of São Paulo (USP), São Carlos 13563-120, SP, Brazil
[3] Department of Physiological Science, Piracicaba Dental School, University of Campinas—UNICAMP, Piracicaba 13414-903, SP, Brazil
[4] Fraunhofer Institute for Manufacturing Technology and Advanced Materials IFAM, Wiener Straße 12, 28359 Bremen, Germany
* Correspondence: klaus.rischka@ifam.fraunhofer.de

Supplementary Materials

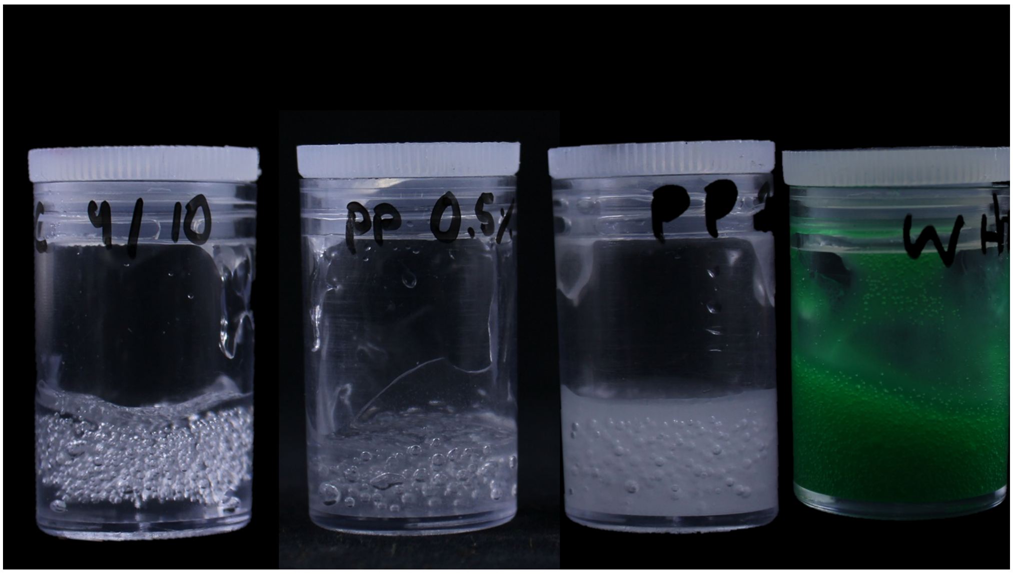

Figure S1. Bleaching gels appearance after manipulation from left to right: Experimental; 0.5% CaPP; 1.5% CaPP; Commercial.
